# Phosphopeptides P140 cause oxidative burst responses of pulmonary macrophages in an imiquimod-induced lupus model

**DOI:** 10.1186/s43556-023-00149-9

**Published:** 2023-11-03

**Authors:** Jianghong Zhong, Chanyu Zheng, Zhongheng Chen, Hangqi Yue, Haiqiang Gao, Yunfan Jiang, Hui Hui, Jie Tian

**Affiliations:** 1 School of Engineering Medicine, Beihang University, Beihang University, No.37 Xueyuan Road, Beijing, 100191 China; 2 Key Laboratory of Big Data-Based Precision Medicine (Beihang University), Ministry of Industry and Information Technology, Beijing, 100191 China; 3 School of Biological Science and Medical Engineering, Beihang University, Beijing, 100191 China; 4 CAS Key Laboratory of Molecular Imaging, Beijing Key Laboratory of Molecular Imaging, the State Key Laboratory of Management and Control for Complex Systems, Institute of Automation, Chinese Academy of Sciences, Beijing, 100190 China

**Keywords:** Lupus, ROS, H3cit, Neutrophils, Macrophages

## Abstract

Recent studies challenge the dogma that a 21-mer phosphopeptide P140 protects against direct cell damage in the phase-III clinical trial (NCT02504645) for lupus, involving reactive oxygen species (ROS)-dependent release of citrullinated histone H3 (H3cit)-linked neutrophil extracellular traps. An open question is the cellular location of ROS production and H3cit formation in lupus. In this study, we examined the effects of P140 peptides on ROS production and H3cit location in lupus with in vivo and situ fluorescence imaging with subcellular resolution. We developed a mouse model of the B6 strain harbouring a bioluminescent reporter under the control of the Lysozyme M promoter. Based on the imiquimod-induced disease model of B6 mice, we used bioluminescent imaging, flow cytometry analysis, and immunohistology staining to study the effects of P140 peptides in lupus. We found a profound accumulation of CX3CR1-positive macrophages in the lungs of lupus mice after the application of P140, accompanied by lung fibrosis formation. The defined P140-mediated macrophage responses were associated with an increase of H3cit in the cytosol, interleukin-1 receptor type 1 on the extracellular membrane, and intracellular production of ROS. Of interest, the disease of imiquimod-induced lupus was prevented with an antioxidant drug apocynin. This study shows that P140 peptides play a role in aggravated murine lupus in a manner dependent on ROS production and H3cit upregulation through pulmonary macrophages.

## Introduction

Systemic lupus erythematosus is an immune-mediated inflammatory disease that may manifest in the kidney and lungs, of which the global prevalence and incidence were around 43.7 per 100 000 persons and 5.14 per 100 000 person-years, respectively [1]. The cause of lupus is not well understood, no cure yet, whereas the use of hydroxychloroquine, cyclophosphamide, and biologics (belimumab) is usually recommended for lupus patients [2]. Single nucleotide polymorphisms in the *Ncf1* gene may predispose to the lupus disease [3], due to the phagocyte NADPH oxidase complex defects in generation of reactive oxygen species (ROS). The *Ncf1* polymorphism was involved in the release of citrullinated histone H3 (H3cit)-linked neutrophil extracellular traps, suggesting an impaired clearance of H3cit^+^ neutrophils by macrophages [4, 5].

Much effort has directed to identify peptide antigens that may trigger the ROS release and restore macrophage clearance for lupus therapy, which might be conferred by major histocompatibility complex (MHC) class II signaling [6]. A popular antigen was the 70-kDa subunit of the U1 small nuclear ribonucleoprotein (U1-70 k) in autoimmune diseases. U1-70 k has a lupus-associated domain containing oxidative cleavage sites [7, 8]. Based on the residues 131–151 at the same domain of this U1-70 k protein harboring the major epitopes as targets of autoreactive CD4 T cells in patients with lupus, the 21-mer synthetic peptide P140 was found protective in lupus-prone MRL-lpr mice and clinical trials by Muller and colleagues [9]. The research group has recently submitted the plan for a new phase 2/3 adaptive clinical trial in lupus to U.S. Food and Drug Administration, but they remained on track in 2023 [10]. Of importance, they reported that neutrophil extracellular traps formation was prevented from fresh human neutrophils after incubation of P140 peptides, measured by using fluorescent intensity of Sytox Green nucleic acid staining [11]. Although the historic view of neutrophils has traditionally been used to evaluate the protective effect of P140 in lupus, however, it remains unclear for the role of P140 how to mediate ROS production by neutrophils and macrophages in control of autoimmune responses.

A recent report provides an alternative narrative to the role of MHC-II positive macrophages in lupus. In contrast to a protective role of macrophages by releasing ROS [2, 4], it was shown as an important player for MHC-II^+^ macrophages to drive lupus nephritis in C57BL/6 (B6) mice treated with topical imiquimod (IMQ), and the hypothesis was validated within monocyte transfer experiments [12]. Thus, the murine model of IMQ-induced lupus provides a unique approach to clarify whether the therapeutic effects of P140 peptides are associated with macrophages. Of interest, using B6 mouse strain expressing bioluminescent reporter protein under the control of the Lysozyme M (Lyz2) promoter [13], the advanced single-cell bioluminescence imaging of deep tissue make it possible to study the role of macrophages in vivo [14, 15]. In addition, application of antioxidant drug apocynin as usual is an option to study oxidative responses in preclinical confirmation research and clinical trials [16]. Therefore, combination of both the IMQ-induced lupus model and non-invasive imaging techniques could provide new insight into P140-mediated effects in a macrophage-dependent manner.

In short, we aimed to study the role of P140 peptides in two distinct mouse models of lupus. We used the commonly used lupus-prone mice of B6.lpr strain, confirming the protective effectiveness of P140 in regulating neutrophil responses. In contrast, using the IMQ-induced lupus model, we observed a profound effect of P140-induced macrophage responses in the lungs with bioluminescence imaging. The P140-mediated effect was associated with both intracellular upregulation of H3cit and extracellular release of ROS by the local macrophages, but it did not affect the pattern of kidney histological changes in the IMQ-induced lupus. Overall, this study shows that P140 may play different roles in the therapy of heterogeneous lupus through ROS signaling derived from neutrophil and macrophages, respectively.

## Results

### Application of P140 led to a more profound accumulation of myeloid cells in lupus

**Fig. 1:**
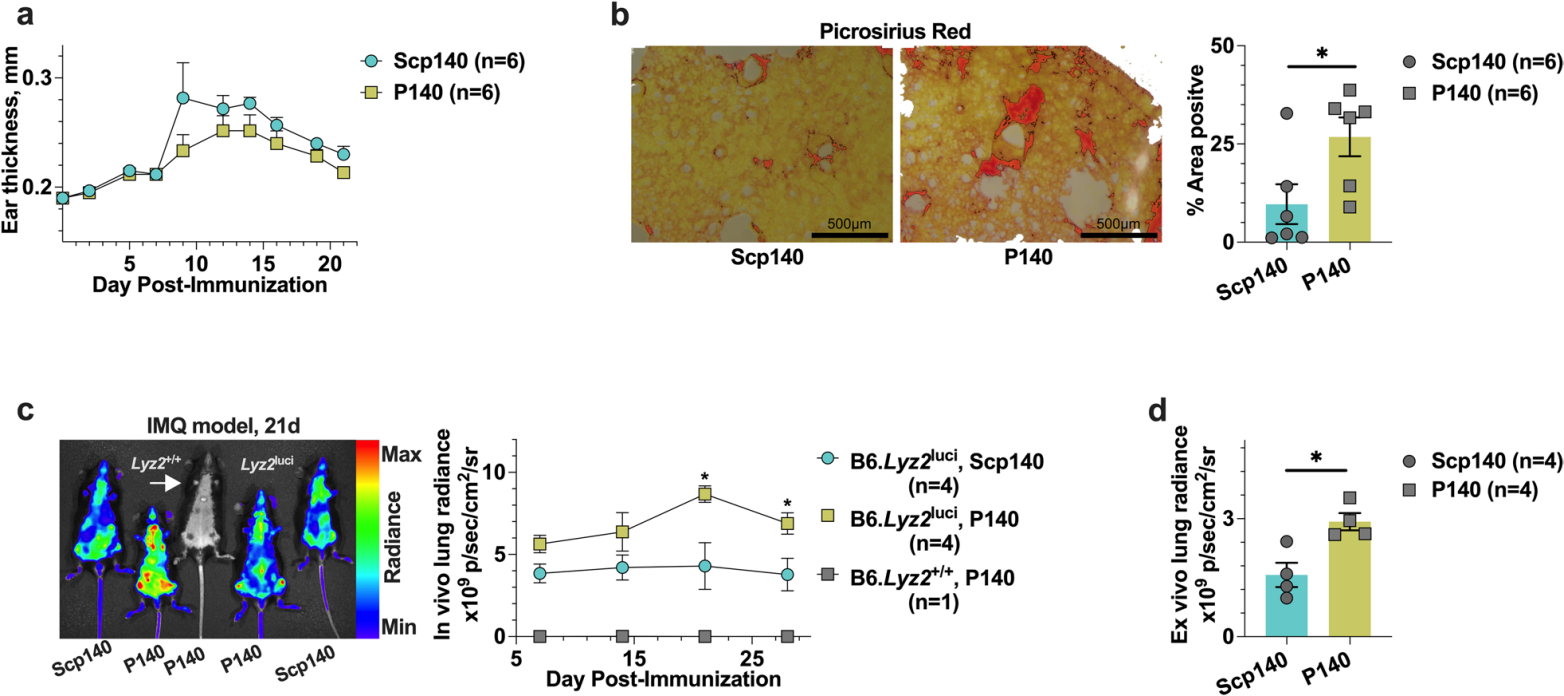
The lung was heightened with P140-mediated responses in the IMQ-induced lupus mice. **a** The ear thickness was measured in the imiquimod (IMQ)-induced lupus model, after B6 mice received the treatments of P140 and Scp140 (6 mice per group). **b** Representative images of picrosirius red staining, and percentage area positive of picrosirius red stained lung sections that were harvested on day 21 after the initial application of IMQ. **c** Representative graph of mice scanned by bioluminescence imaging, showing in vivo optical radiance in the lung region along with the disease development in B6.*Lyz2*.^luci^ mice received P140 and Scp140 (4 mice per group), compared with a wild type mouse. **d** Optical radiance of the tissue samples, prepared from the lungs, spleens, and kidneys in mice on day 28 after the initial application of IMQ. The *p* values determined in the Mann–Whitney test were shown as *p < 0.05

To identify the tissue-selective manifestations in lupus, we studied the effect of P140 peptides in the IMQ-induced lupus. Using B6 mice with the IMQ-induced lupus, we monitored the murine ear thickness change (Fig. 1a), and two groups of mice exhibited a similar response after the treatments of P140 peptides and the phosphorylated peptide Scp140 used as controls. Based on the B6 mouse model, we harvested the tissue samples on day 21 after the initial application of IMQ, and performed the picrosirius red staining to analyze quantitatively the degree of fibrosis formation as shown in a representative graph (Fig. 1b). Interestingly, percentage-area positive of the picrosirius red staining increased after treatments of P140 (Fig. 1b). To the noninvasively demonstrate the effects of P140 peptides with the advantage of molecular imaging techniques, we used the IMQ-induced lupus model of B6*.Lyz2*^luci^ mouse strain, monitoring the spatiotemporal distribution of bioluminescent neutrophils and macrophages in the whole body. Figure 1c shows a representative graph, we observed the optical radiance emitted from myeloid cells in vivo. Application of P140 peptides resulted in the dominant radiance occurring near the lung region of interest along with the disease development (Fig. 1c). At the end point of experiments, i.e., on day 28 after the initial application of IMQ, to exclude the potential effects from the periphery blood cells, we performed cardiac perfusion with 10 mL ice-cold PBS, collected the tissues samples of the lungs, and verified the bioluminescent radiance of myeloid cells (Fig. 1d). The optical radiance shows that administrations of P140 led to an increase in the lung macrophage activity in lupus (Fig. 1d). Thus, our results reveal that P140 could promote circulating myeloid cell migration into the lungs and surrounding spaces.

### Application of P140 led to splenic macrophage expansion in lupus

**Fig. 2:**
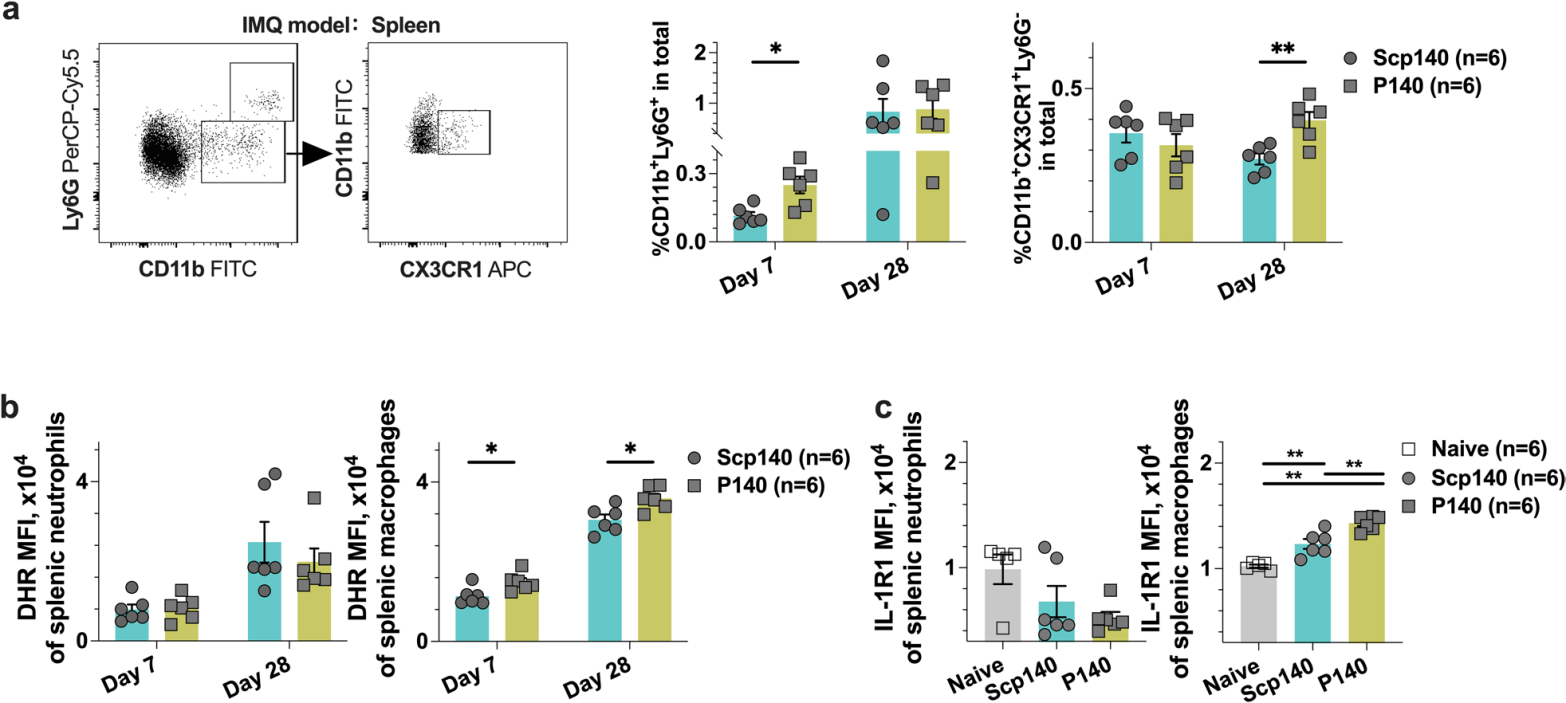
Splenic macrophage expansion was present in the IMQ-induced lupus mice with P140 treatments. B6 mice received the treatments of P140 and Scp140, compared with the naïve mice (6 mice per group). The tissue samples were harvested on day 7 and day 28 after the initial application of IMQ. **a** A representative flow cytometric graph of splenocytes, and the frequencies of neutrophils (CD11b^+^Ly6G^+^) and macrophages (CD11b^+^CX3CR1.^+^) in the spleen. **b** The MFI of DHR staining, and, **c** the MFI of IL-1R1 expression in splenic neutrophils and macrophages, respectively. The *p* values determined in the Mann–Whitney test were shown as *p < 0.05 and **p < 0.01

To clarify the cell type of circling myeloid cells migration in lupus with P140 treatments, we studied the change of splenocyte frequency pattern that is an essential player in B6 mice with the IMQ-induced lupus. At the end point of experiments, we harvested the splenic samples for quantitative assessments. The cells were analyzed using flow cytometry, and the dot plots show the selected windows and gating strategy as applied to the identification of splenic neutrophils and macrophages (Fig. 2a). In lupus mice treated with P140 peptides after the initial application of IMQ, we observed an increased proportion of splenic neutrophils on day 7, and increased proportion of splenic CX3CR1^+^ macrophages on day 28 (Fig. 2a). Detections of intracellular ROS in neutrophils, indicated as the mean fluorescence intensity (MFI) of DHR staining, were found similar between two groups of mice (Fig. 2b). In contrast to neutrophils, CX3CR1^+^ macrophages had the higher content of ROS production in P140-treated mice than that of Scp140-treated mice, based on the detections of ROS on day 7 and day 28 after the initial application of IMQ (Fig. 2b). Interestingly, we found an increase in IL-1R1 expression from CX3CR1^+^ macrophages, which was consistent with the intracellular ROS status on day 28 after the initial application of IMQ (Fig. 2c). Thus, based on the IMQ-induced lupus model, we found an intrinsic increase in IL-1R1 expression and extrinsic release of ROS in CX3CR1^+^ macrophages responses to P140 treatments.

### Application of P140 resulted in an accumulation of the lung macrophages for lupus mice

**Fig. 3:**
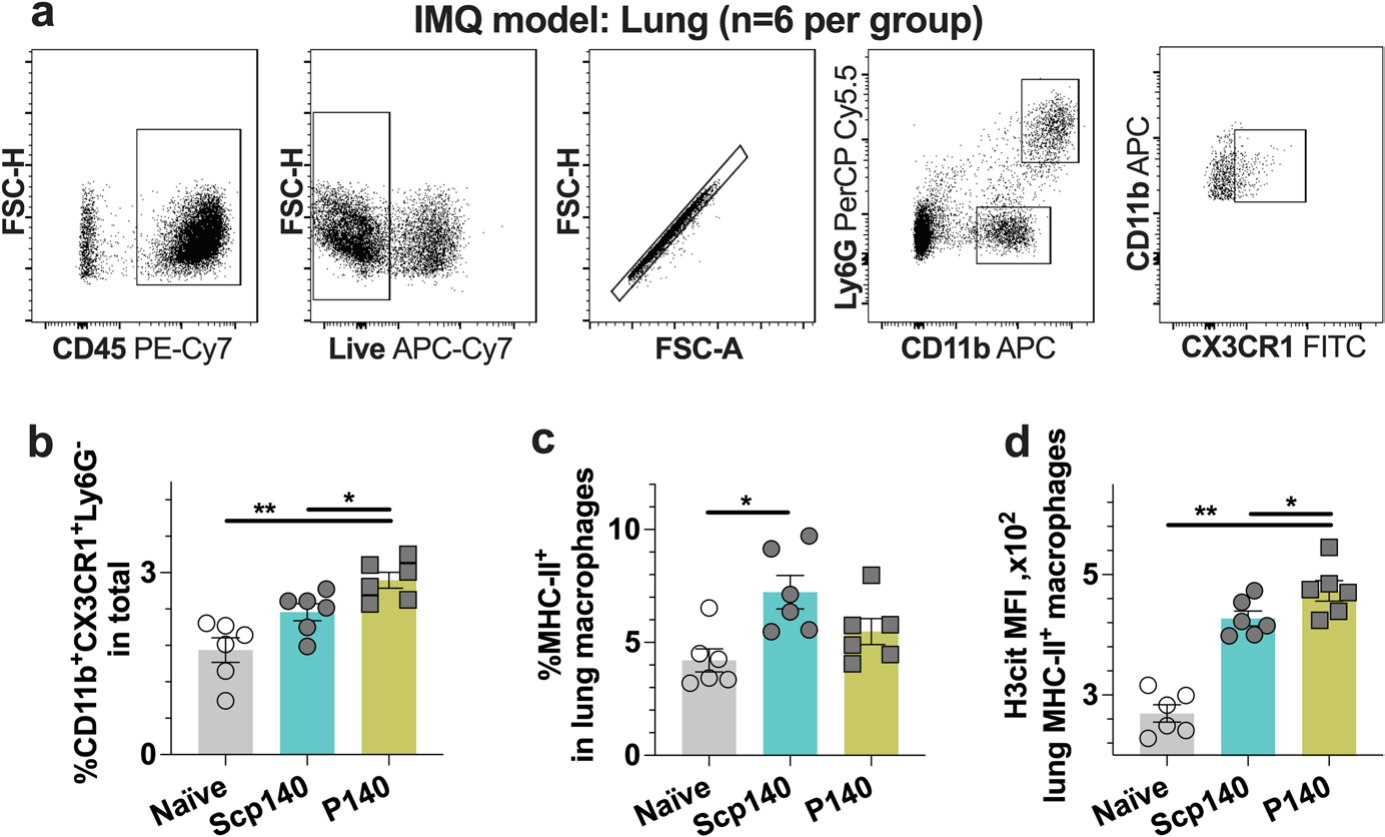
Flow cytometry analysis shows upregulation of H3cit in lung macrophages in vivo. B6 mice received the treatments of P140 and Scp140 (6 mice per group). The tissue samples were harvested on day 21 after the initial application of IMQ. **a** A representative flow cytometric graph of splenocytes, and the frequencies of neutrophils (CD11b^+^Ly6G^+^) and macrophages (CD11b^+^CX3CR1^+^) in the lung. **b** The frequencies of macrophages (CX3CR1.^+^) in the lungs and, **c** the frequencies of MHC-II positive cell in the lung macrophages. **d** the MFI of H3cit staining in MHC-II positive macrophages. The *p* value determined in the Mann–Whitney test was shown as *p < 0.05 and **p < 0.01

To clarify the cell type of accumulated myeloid cells into the lungs, we studied the change of the tissue-resident macrophage frequency pattern in lupus mice with flow cytometry analysis. We harvested the tissue samples on day 28 after the initial application of IMQ. We measured the ROS-mediated H3cit expression upon the principle of a gating strategy for myeloid cells (Fig. 3a). The proportion of lung macrophages increased in the lupus mice after P140 treatments (Fig. 3b), which was consistent with both in vivo and ex vivo measurements of optical radiance with bioluminescence imaging. The MHC-II expression was similar on lung macrophage surface for the lupus mice treated with peptides of P140 and Scp140 (Fig. 3c). Interestingly, treatments of P140 promoted H3cit protein expression in the lung macrophages (Fig. 3d). H3cit-positve macrophages may be one of autoimmune disorders with increased efferocytosis and clearance failure of neutrophil extracellular traps. Thus, the results suggest P140-induced H3cit enhancement may result from clearance failures and delay degradation of CX3CR1^+^ macrophages in lupus.

### Application of P140 resulted in the cytosol H3cit generation of the lung macrophages

**Fig. 4:**
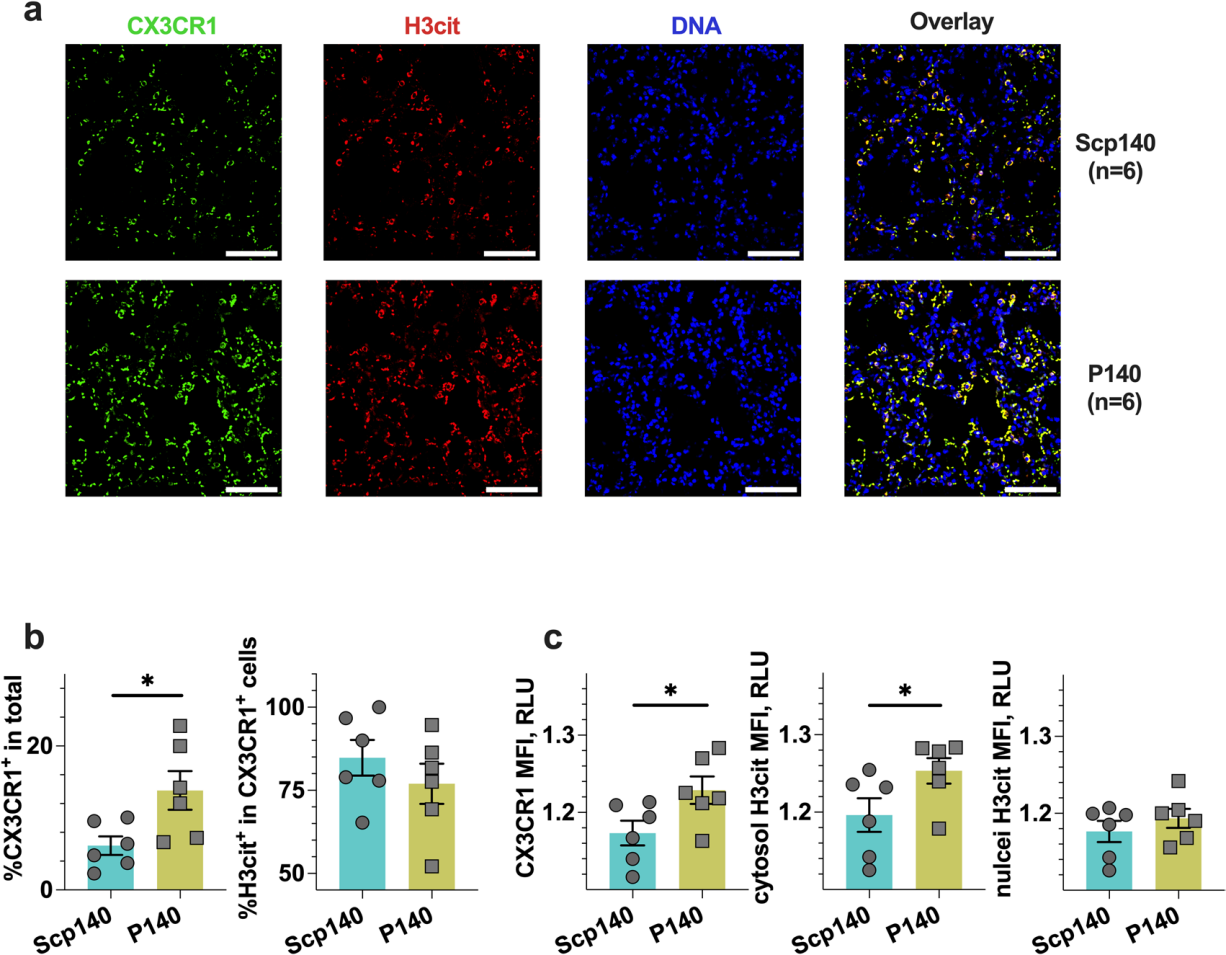
Immunohistochemistry profile reveals a cytosol H3cit formation of macrophages in vivo. B6 mice received the treatments of P140 and Scp140 (6 mice per group). The tissue samples were harvested on day 21 after the initial application of IMQ. **a** Representative images of the lung CX3CR1, H3cit, and DNA staining; scale bar, 50 μm. **b** The frequencies of macrophages (CX3CR1^+^) in the lungs, and the frequencies of H3cit positive cell in the lung macrophages. **c** the MFI of CX3CR1 and H3cit staining in macrophages. The p value determined in the Mann–Whitney test was shown as *p < 0.05

To identify subcellular signature of CX3CR1^+^ macrophages in the lungs, we studied the change of the tissue-resident macrophage profile pattern in lupus mice with immunohistochemistry profile analysis. We harvested the tissue samples on day 21 after the initial application of IMQ. Taking the advantage of microscope imaging as shown in a representative graph (Fig. 4a), we observed a more profound infiltration of CX3CR1^+^ macrophages in the lungs (Fig. 4b). Of note, the colorful readouts of immunofluorescence microscopy enabled us to perform image segmentation processing and subsequently analyze single-cells with a sub-cellular resolution, whereas we observed an increase in H3cit MFI in the cytosol but not in the nucleus of the local macrophages upon P140 treatments (Fig. 4c). Thus, the results suggest P140-induced cytosol enhancement of H3cit in macrophages may be associated with the rarely pulmonary fibrosis in lupus.

**Fig. 5:**
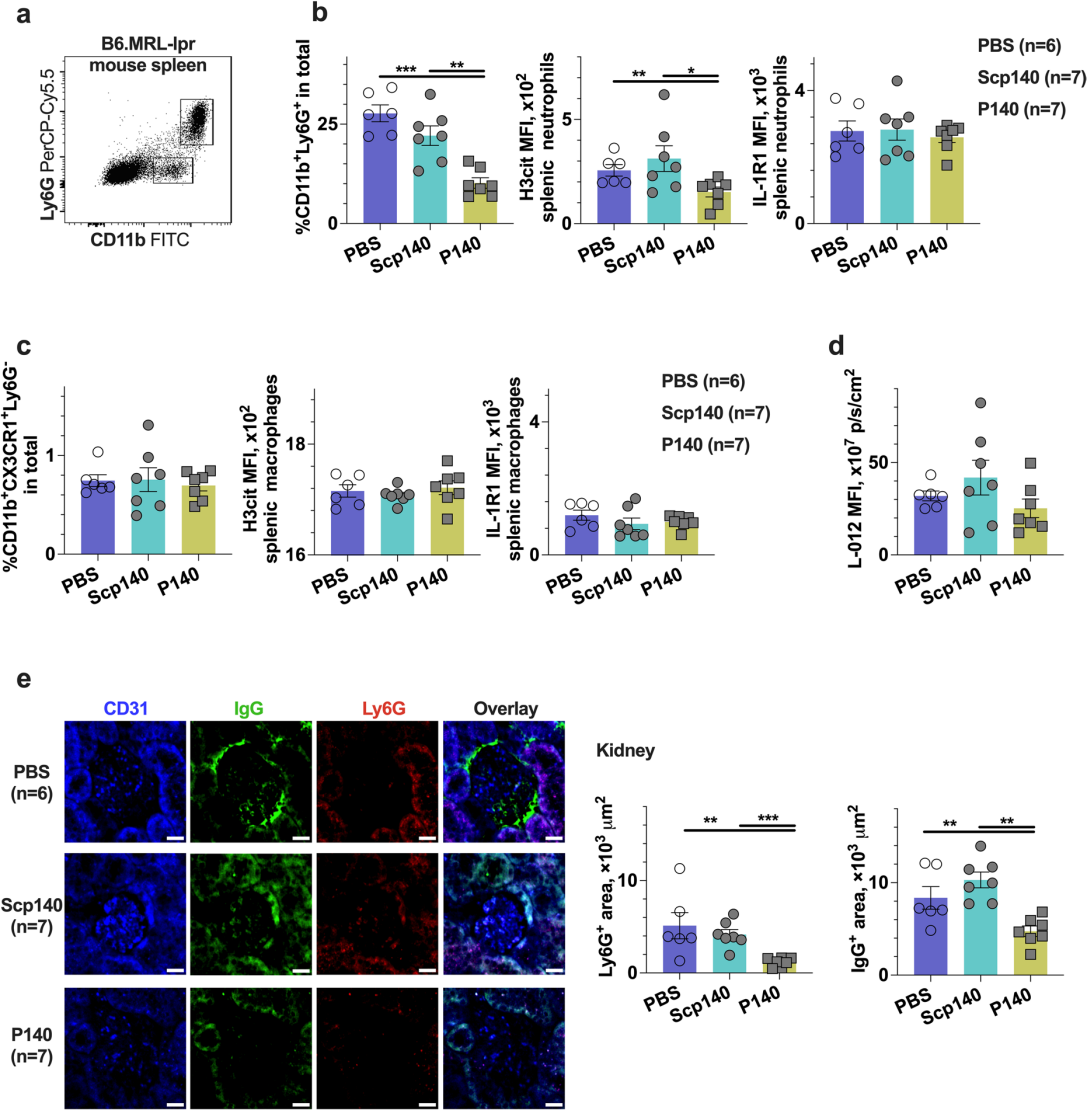
The P140 treatments protected against neutrophilia in the lupus-prone mice. B6.lpr mice received P140 (7 mice), Scp140 (7 mice), and PBS (6 mice). The spleen samples were harvested on day 28. **a** Representative graph of splenic neutrophils (CD11b^+^Ly6G^+^). **b** The frequency of neutrophils in the spleen, as well as mean fluorescence intensity (MFI) of targeted staining. **c** The frequency of macrophages (CD11b^+^CX3CR1^+^Ly6G.^−^) in the spleen, and MFI of targeted staining. **d** MFI of optical radiance by performing the L-012 chemiluminescence assay with the fresh splenocytes in responses to PMA stimuli ex vivo. **e** Representative images of the kidney CD31, Ly6G, and IgG staining (scale bar, 50 μm), and area sizes of the kidney Ly6G and IgG positive staining. The *p* values determined in the Mann–Whitney test were shown as *p < 0.05, **p < 0.01, and ***p < 0.001

Additionally, we used the same peptides of P140 to repeat the previous experiments, confirming the protection of P140 peptides against the neutrophil extracellular trap formation in lupus-prone B6.lpr mice [11]. In this study, B6.lpr mice received intraperitoneal administrations of P140 and Scp140 for 4 weeks, whereas the dose of peptides was 100 μg for each mouse every week. At the end point of experiments, we prepared the splenic samples for quantitative assessments with flow cytometry. In the mouse model, P140 reduced the frequency of neutrophils in the spleen (Fig. 5a), of which H3cit expression was suppressed (Fig. 5b). We verified that P140 did not change either the proportions of splenic macrophages or the pattern of macrophage H3cit expression in lupus mice (Fig. 5c). The ROS production of splenocytes was also evaluated by performing the L-012 luminescence assay in response to PMA stimulation ex vivo. We verified that P140 did not change ROS production (Fig. 5d). To further confirm the effect of P140 peptides in lupus-prone B6.lpr mice, we screened the change of the neutrophil location in the kidneys. The immunofluorescence results indicate a downregulation of both neutrophil infiltration and IgG expression after P140 treatments (Fig. 5e). Thus, this study shows that P140 ameliorated the disease development in lupus-prone mice, associated with a reduce in H3cit positive neutrophils, suggesting that P140 plays different roles in two models of lupus.

### ROS blockade prevented from P140-mediated macrophage responses in lupus

**Fig. 6:**
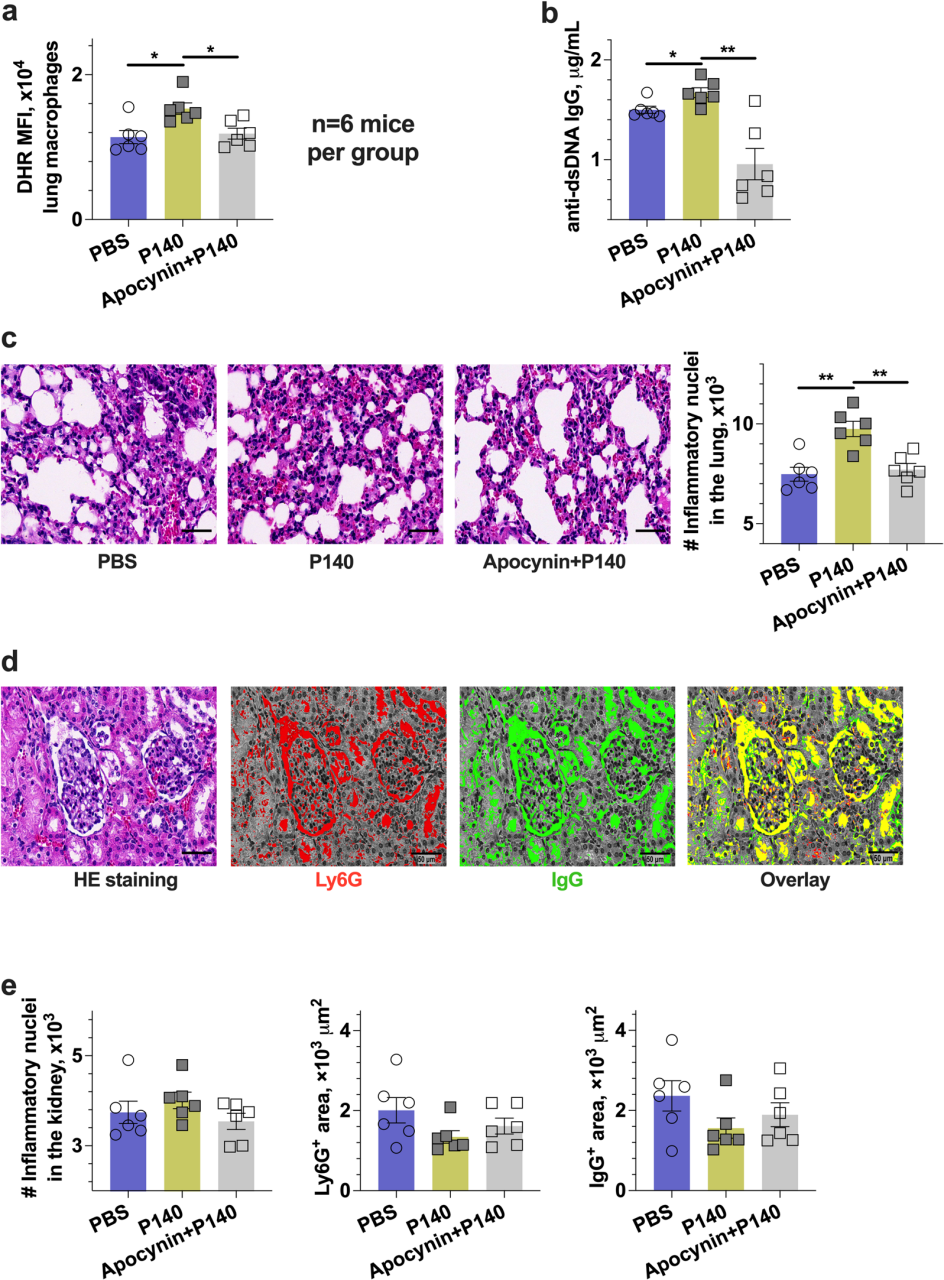
The P140-mediated lung inflammation was prevented by an antioxidant in the IMQ-induced lupus mice. B6 mice received P140 and Apocynin, compared with PBS (6 mice per group). Samples were harvested on day 28 after the initial application of IMQ. **a** The MFI of DHR staining in lung CD11b^+^Ly6G^−^CX3CR1.^+^ monocytes and macrophages. **b** The titration of serum anti-dsDNA IgG antibodies. **c** The representative images of the lung sections by HE staining, and the number of inflammatory nuclei in the lungs; scale bar, 50 μm. **d** Representative images of the kidney sections by HE staining, and the predictive distribution maps of anti-mouse Ly6G and IgG staining; scale bar, 50 μm. **e** The number of inflammatory nuclei, and area size of Ly6G and IgG positive staining in the kidney section, respectively. The *p* values determined in the Mann–Whitney test were shown as *p < 0.05 and **p < 0.01

To demonstrate the role of ROS in macrophage responses to P140 treatments, we evaluated the disease course of lupus by applying an antioxidant apocynin. We harvested the samples of the lung, kidney, and serum from each induvial on day 28 after the initial application of IMQ. The effect of ROS blockade was examined by performing the DHR assay in response to PMA stimulation ex vivo, in which ROS production was inhibited for the lung macrophage subsets (Fig. 6a). In the mouse model, the serum IgG anti-dsDNA antibody was detected. Of interest, with the combined therapy of P140 peptides and apocynin, IgG anti-dsDNA antibodies were decreased in lupus mice (Fig. 6b). Thus, we examined the histopathological changes in the tissue sections by performing the HE staining assay. We found the inflammatory nuclei number reduced in the lung sections after the combined application of P140 and apocynin (Fig. 6c). However, we failed to find the histological changes of HE stanning in the kidney sections (Fig. 6d and e). Although proteinuria score was determined with semiquantitative urine testing strips (Albustix, Bayer, China) using midstream urine, there was also no difference observed across three groups of mice. Thus, these results show that combined application with antioxidants may suppress the local macrophage response to peptide-alone therapy in lupus.

## Discussion

We observed that treatments of P140 promoted the oxidative burst response by pulmonary macrophages in the IMQ-induced lupus model, showing a more profound accumulation of H3cit-positive macrophages in the lungs and the potential fibrosis formation.

It makes a challenge to non-invasively address the role of P140 peptides due to the etiology of heterogenous lupus. The lupus patients usually carry anti-U1-70 k antibodies sharing the conserved epitopes with mouse models [8, 17]. A list of such epitopes has been studied with synthetic peptides to probe the causal factors for improving lupus therapy. Using a 142-amino-acid motif of U1-70 k containing an immunodominant epitope with P140 peptides, Hoffman and colleagues observed the lung disease of B6 mice [18]. Using P140 peptides through different injection methods, Muller and colleagues demonstrated that the subcutaneous injection accelerated the development of experimental glomerulonephritis in MRL-lpr mice, while the intravenous injection delayed the disease development of MRL-lpr mice [9]. In this study, we used intraperitoneal injections of synthetic peptides P140 encompassing the same sequence as Lupuzor™ to two distinct lupus models. In the lupus-prone B6.lpr mice, we examined neutrophil extracellular traps generation in lupus measured by H3cit expression [11, 19]. Our study confirmed that treatments of P140 protected against lupus nephritis with neutrophil infiltration suppression, showing reduced H3cit in neutrophils (Fig. 5b). However, using P140 in the IMQ-induced lupus model, we found a highlight of the lung macrophage-mediated response with picrosirius red staining (Fig. 1b) and bioluminescent imaging (Fig. 1c and d). Furthermore, we performed the flow cytometry analysis of splenocytes (Fig. 2) and the lung samples (Fig. 3), respectively. In the IMQ-induced lupus model, we observed an expansion of myeloid cells in the spleens, such as neutrophils in the very early stage (day 7, Fig. 2a) and, macrophages in the advanced stage of disease development (day 28, Fig. 2a). We did not observe a detectable change of ROS production and H3cit stanning for neutrophil responses to P140 treatments, but found the change occurring in splenic macrophages based on the measurements of splenocytes and the kidney stanning in the IMQ-induced lupus model (Fig. 2b). Of importance, we found H3cit increase in lung macrophages at the late stage of lupus therapy (day 28, Fig. 3d), which may exacerbate pneumonia in the induced lupus following P140 treatments. It can cause tissue damage for ROS as an initial effector in macrophage responses [20], although the underlying mechanisms are not understood yet.

The previous studies reported the interactions between macrophages and autoreactive T cells through paracrine mechanisms [21–25]. More recent studies showed that ROS was also involved in intracellular process of macrophages [26], as well as chaperone-mediated autophagy and lysosomal storage disorders [27]. The Lyz2-Cre-mediated deletion of NOX2 activity resulted in upregulation of macrophage IL-1R1 expression, spontaneous change in the transcriptome and epigenome of alveolar macrophages, and increased responses to Toll-like receptor-2 (TLR2) and TLR4, whereas the low-level development of lung inflammation did not require host microbiota or the type I IFN receptor [26]. The heterogeneous pattern of lung inflammation may start with autocrine up-regulation of macrophage IL-R1 expression in lupus-prone mice [28–30]. Of interest, IL-1R1 deficiency almost abolished the IMQ-induced disease, but the disease was not affected by defects with IL-1 alpha and beta [31]. Therefore, we studied the ROS production and IL-1R1 expression in both spontaneous and induced lupus models. Based on the IMQ-induced lupus model with B6 mice, we found that the lung macrophages had the memory of enhanced ROS production when performing ex vivo assays (Fig. 2b), as known as the high robustness of the lung macrophages restoring their identity [32]. In the above two groups of lupus mice, we found that P140 treatments led to an increase in IL-1R1 and H3cit of macrophages (Fig. 2c). What it is more, we detected a lower level of IL-1R1 in macrophages from B6.lpr mice than that in B6 mice (Fig. 5b and c). Interesting, we used an antioxidant drug apocynin (Fig. 6a), which almost abolished the onset of chronic lung injury that might be induced by P140 treatments (Fig. 6b and c). Our results suggest that the generation of ROS was required for the lung macrophage responses upon applications of P140 peptides. However, the short-live ROS make it difficult to monitor its contribution using conventional methods. Combining single-cell RNA sequencing and spatial transcriptomics may separate macrophage subsets and reveal the complexity of P140-mediated oxidative responses in lupus. It will be interesting to further address the cell-extrinsic factor P140 related to the lung niche though the TLR2/4 and IL-1 pathways. Overall, our results suggest an autocrine mechanism underlying the lung inflammation mediated by macrophages through IL-1R1 signaling.

The H3cit macrophages may cause the lupus-like disease, due to defective clearance of neutrophil extracellular trap [12, 20, 33]. The H3cit is rich during neutrophil extracellular trap formation, which is to be degraded by macrophages [34, 35]. However, macrophages either did not produce proinflammatory cytokines or did not promote inflammation after ingestion of neutrophil extracellular traps alone [36, 37]. Therefore, it is an issue to identify the tissue-resident macrophages with a non-invasive technique [13, 15]. In this study, we observed that P140 treatments were accompanied with a more profound formation of lung fibrosis, although the interstitial expansion and tubular dilation could be present in the lung tissues of the IMQ-induced lupus mice (Fig. 1b). Based on the lupus model of B6 mice, we used molecular imaging technique with the advantage of high sensitivity to verify the bright bioluminescence near the lungs (Fig. 1c). The results were consistent with between small animal imaging and flow cytometry measurements, wherein P140 treatments led to the expansion of H3cit^+^ macrophages and lung inflammation in the IMQ-induced lupus (Fig. 3c and d). Furthermore, we clarified the immunohistochemistry profile and subcellular location of enhanced H3cit in lupus mice with P140 treatments. Enhancement of H3cit was observed in the cytosol, but not in the nucleus of the tissue-resident macrophages (Fig. 4a and c). Thus, our results suggest P140-mediated H3cit macrophage responses were associated with pulmonary manifestations in lupus, which could prognostically resemble interstitial lung diseases (Fig. 4). However, due to the limitations of subcellular structure-based protein function in this study, it will be interesting to address the roles of H3cit-positive macrophages through the phagocyte NADPH oxidase complex-derived ROS release, apoptotic cell clearance, cytosolic DNA sensing and type I interferon production, to identify the effects of P140 peptides in lupus at details, and to exclude other types of lung involvement such as pulmonary vascular disease, bronchiolitis, and other airspace abnormalities in the next future.

In summary, this study dissects the roles of P140 peptides in two distinct lupus models with small animal imaging. The cell-intrinsic factors of pulmonary macrophages, i.e., both a cytokine receptor IL-1R1 on the plasma membrane, and the key intracellular molecule H3cit, may couple with the cell-extrinsic factors to drive the development of low-level lung inflammation. P140 used as a potential drug could provide the extrinsic contribution to the low-level chronic inflammation through macrophage responses.

## Materials and methods

### Animal model and genotyping

Founders of B6 mice (C57BL/6 J) and B6.lpr mice (B6.MRL-*Fas*^lpr^/J) are from the JAX Lab (Maine, US). B6*.Lyz2*^luci^ mice (Stock No: NM-KI-200029) are bought originally from Shanghai Model Organisms Center (Shanghai, China). B6*.Lyz2*^luci^ mouse strain has a knock-in of the Akaluc luciferase (luci) reporter gene after the stop codon of the endogenous *Lyz2* gene using CRISPR. The specific-pathogen free mouse strains are maintained by the Zhong laboratory (Beijing, China). The primers for *Fas*^lpr^ genotyping are as follows: 5’-GTAAATAATTGTGCTTCGTCAG-3’ (common), 5’- TAGAAAGGTGCACGG GTGTG-3’ (mutant), 5’-CAAATCTAGGCATTAACAGTG-3’ (wild-type). The primers for *Lyz2*^luci^ genotyping are as follows: 5’-GAGGAGGCAGAGCATCAAAC-3’ (mutant forward), 5’-ACAAACGCACACCGGCCTTATTCC-3’ (mutant reverse), 5’-TTTTATCCATTTAACCCCATGTCTCTTTCC-3’ (wild-type forward), 5’- CACAAAGCCCTGCAGCTCACACGAC-3’ (wild-type reverse). We performed the experiments with 8- to 9-week-old female mice. The co-housing and littermate methods were used under specific pathogen-free conditions. The identity of experimental mouse was blinded for the investigator, and each mouse weighed approximately 20 g.

### Antibodies

The antibodies used in current study were shown previously, accompanied with the flow cytometry staining protocol [19]. The other antibodies included CX3CR1 (clone: SA011F11, APC, BioLegend), CD68 (clone: FA-11, FITC, BioLegend), Ki67 (clone: 16A8, APC, BioLegend), CD3ε (clone: 145-2C11, PerCP/Cy5.5, BD Biosciences), CD4 (clone: RM4-5, FITC, BD Biosciences), MHC-II Ab (clone: AF6-120.1, FITC, BD Biosciences), IL-1R1 (clone: 35F5, PE, BD Biosciences).

### Imiquimod induced lupus

IMQ 5% cream (15 mg/mouse, Aldara, Guangzhou, China) was administrated topically on the inner ear in female mice for three times every week. The linear phosphorylated P140 peptide H_2_N-RIHMVYSKRp**S**GKPRGYAFIEY-COOH (L-Tyr at the C-terminus, molecular weight 2639, purity $\ge$ 98%, 100 μg/mouse, GL Biochem, Shanghai, China) were dissolved in PBS. The Scp140 peptide (H_2_N-YVSRYFGp**S**AIRHEPKMKIYRG-COOH, L-Tyr at the N-terminus, molecular weight 2639, purity $\ge$ 98%, 100 μg/mouse, GL Biochem, Shanghai, China) was used as the peptide control in this study. Peptide homogeneity was checked, and the identity was assessed by liquid chromatography-mass spectrometry (GL Biochem, Shanghai, China). Both of these peptide batches were sterile and injected intraperitoneally. Apocynin (2.5 mM, CAS No. 498–02-2, MCE, China) was supplied in drinking water during the treatment period.

### Optical imaging

When mice were anaesthetized, a bioluminescence probe TokeOni (Catalog No. 808350-25MG, Sigma-Aldrich, China) dissolving in sterile water (5 mM) was injected with a dose of 100 $\mu$ L/mouse to detect macrophages with an IVIS ® Spectrum imager (PerkinElmer, Shanghai, China) [13, 38]. For ROS detection in vivo, a chemiluminescence probe L-012 (Catalog No. 5085, Wako, Japan) was used as the previous protocol [19].

### Flow cytometry

The murine samples were prepared from both the lung and spleens using the previous protocol of flow cytometry analysis [19, 39]. Before the single-cell suspension analysis, the lung fragments dissected from each mouse sample were incubated in PBS for 45 min, together with 2 mg/mL collagenase type IV (CAS No. 9001–12-1, Sigma, China), 100 U/mL DNase I (Catalog No. 04716728001, Roche, China), and 0.5 mM EDTA (CAS No. 60004, Sigma, China).

### Enzyme-linked immunosorbent assay

Murine sera were added at a 1:100 dilution in PBS plus 2% FBS (Gibco, CAS No. 10099–141, Australia). The microtiter plates were precoated with 20 μg/ml poly-L-lysine (CAS No. 25988–63-0, Sigma-Aldrich, China) before adding 20 μg/ml S1 nuclease-treated calf thymus DNA (CAS No. 73049–39-5, Sigma-Aldrich, China). Bound IgG was detected with 0.125 μg/ml horseradish peroxidase–conjugated goat anti–mouse IgG(H + L) (Catalog Number 1030–5, Southern Biotech, US) and substrate solution. The samples were scanned with the 450 nm filter by a 96-well plate reader (Bio-Rad iMark, US).

### Picrosirius red staining assay

Paraffin-embedded formalin-fixed Sects. (40-μm thick) were stained with 0.1% picrosirius red (CAS No. 14726–29-5, Sigma-Aldrich, China) following a standard protocol. In chief, each tissue slice was covered with 50 μL of picrosirius red dye for 2.5 h. Images of picrosirius red staining of lung tissue sections were quantitatively analysed using the Imaris version 9.3.0 (Bitplane AG, UK).

### Histology

Mice were sacrificed at the end of the lupus experiment. Both the left lung superior lobe and the left kidney per mouse were collected and fixed for 5 days in 4% formaldehyde (CAS No. 100496, Sigma-Aldrich, China), decalcified with ethylenediaminetetraacetic acid (EDTA, CAS No. 60004, Sigma-Aldrich, China), and embedded in paraffin (CAS No. 8002–74-2, Sigma-Aldrich, China). Tissue sections were stained with HE, scanned by the microscope, and assessed with the mice identity blinded for the investigator. The inflammatory nuclei were counted according to HE staining.

### Immunofluorescence

The mice were sacrificed at the end of the experiments to harvest the tissue samples. The samples of the lungs and kidneys were incubated for 7 days in 4% paraformaldehyde (CAS No. 30525–89-4, Sigma-Aldrich, China), and then embedded in 1% agarose (MoreBetter Biotechnology, Catalog No. AgarPowder1400, China). We prepared the tissue sections without specific lesions with a sample thickness of 40 μm for immunostaining. The sections at 20 °C up to 4 h were blocked with 1% FBS in 1 × PBS, stained with fluorescently conjugated antibodies (the lungs: CD11b, CX3CR1, H3cit; the kidneys: IgG, Ly6G, CD31) for one day at 4 °C, and then washed. Three images were acquired for each slide at 40 × using a microscope (Andor Dragonfly 200, Oxford Instruments, England). The predictive distribution maps of anti-mouse Ly6G and IgG staining was established by the approach of the UNet + + method [40].

### Statistics

We performed the statistical analysis to compare the differences between two independent groups, using the Mann–Whitney U test (GraphPad Prism 9.5.1, San Diego, CA, US). The statistical results are shown as mean ± standard error of the mean (SEM). The *p* value of statistical analysis was shown, such as *p < 0.05, **p < 0.01, and ***p < 0.001.

## Data Availability

The data of this study are available from the corresponding author on reasonable request.
